# Advances in polyphenol-based strategies for musculoskeletal recovery and exercise rehabilitation in cancer: Mechanistic insights into Wnt/β-catenin and PI3K/Akt signaling pathways and inflammatory markers

**DOI:** 10.3389/fonc.2026.1805804

**Published:** 2026-04-01

**Authors:** Yulei Li, Xiujuan Guan

**Affiliations:** 1 School of Psychology, Beijing Sport University, Beijing, China; 2 School of Physical Education, Pingdingshan University, Pingdingshan, Henan, China; 3 College of Foreign Language, Pingdingshan University, Pingdingshan, Henan, China

**Keywords:** cancer, muscle recovery, PI3K/AKT, polyphenols, sport performance, Wnt/β-catenin

## Abstract

Musculoskeletal dysfunction and compromised physical performance are prevalent complications observed in individuals diagnosed with cancer, frequently intensified by the administration of chemotherapy, radiotherapy, and extended periods of physical inactivity. Polyphenols (bioactive constituents derived from various plant sources) have exhibited significant anti-inflammatory, antioxidant, and anti-apoptotic characteristics that may serve to augment exercise interventions aimed at improving musculoskeletal recovery and rehabilitation outcomes. This review encapsulates the existing preclinical and clinical evidence regarding the synergistic effects of polyphenol supplementation in conjunction with structured exercise protocols in the context of cancer, with particular emphasis on underlying molecular mechanisms and functional outcomes. Prominent signaling pathways influenced by these combined therapeutic strategies include Wnt/β-catenin, PI3K/Akt, TNF-α/NF-κB, IL-4/STAT6, and TGF-β1/TRAF6, which are integral to the regulation of inflammation, apoptosis, muscle metabolism, and tissue remodeling. Preclinical investigations conducted using rodent cancer models consistently reveal that polyphenols such as curcumin, resveratrol, and genistein, significantly enhance the efficacy of both aerobic and resistance exercise concerning tumor suppression, muscle preservation, and modulation of molecular pathways. Clinical evidence, although scarce, suggests potential enhancements in muscular strength, endurance, and recovery when polyphenolic compounds are utilized in conjunction with exercise rehabilitation, particularly among individuals undergoing oncological treatment. Despite these encouraging results, variability in dosing regimens, formulations, timing, and participant demographics constrains the generalizability of the findings. Subsequent research endeavors should prioritize the development of standardized polyphenolic preparations, refined exercise protocols, and clinically relevant functional outcomes to establish evidence-based clinical guidelines. The integration of polyphenols with exercise represents an auspicious non-pharmacological approach to augment musculoskeletal function and enhance quality of life in the context of cancer rehabilitation.

## 1 Introduction

Polyphenols are increasingly recognized as a valuable nutritional approach for supporting overall health because they contain bioactive components that extend their benefits beyond basic nourishment. Polyphenols constitute a diverse group of plant−derived bioactive compounds, traditionally categorized into four main classes based on the number of phenol rings and the structural elements that bind these rings: flavonoids, phenolic acids, stilbenes, and lignans. Flavonoids represent the most abundant and widely studied class and are further subdivided into several subclasses including flavonols, flavones, isoflavones, flavanones, flavan−3−ols (catechins), and anthocyanins. Within this hierarchy, flavonols, characterized by the presence of a 3−hydroxyflavone backbone, are prominent dietary constituents, with quercetin being one of the most widely consumed and extensively researched members. Similarly, flavan−3−ols such as catechin, epicatechin, and epigallocatechin gallate (EGCG) are abundant in foods such as tea, cocoa, and certain fruits and have been widely investigated for their physiological effects. These structural distinctions are critical, as the specific chemical configuration of each subclass influences its stability, bioavailability, metabolic pathways, and biological activity, thereby shaping its capacity to modulate oxidative stress, inflammatory signaling, and cellular pathways involved in muscle repair, connective tissue remodeling, and overall musculoskeletal recovery (1).

Polyphenols are defined by their ability to influence physiological processes in ways that may help lower the risk of chronic disorders and enhance general wellness (2). Major health authorities, including EFSA, the FDA, and WHO, recognize the potential role of functional foods and bioactive dietary compounds in promoting health and reducing disease risk (3). Naturally occurring polyphenols are commonly found in fruits, nuts, vegetables, and whole grains. They provide high levels of essential nutrients, fibers, antioxidants, and phytochemicals that support biological resilience (4–7). In contrast, processed functional foods are intentionally fortified, enriched, or reformulated to increase their concentration of beneficial ingredients or to reduce components that negatively affect health (8). Beyond their general health-promoting properties, polyphenols have attracted increasing attention for their potential role in modulating inflammation, oxidative stress, and metabolic pathways that influence musculoskeletal health and recovery.

Dietary nitrates are naturally occurring inorganic compounds abundant in vegetables such as beetroot, spinach, arugula, and lettuce. Following ingestion, nitrates are converted through the nitrate–nitrite–nitric oxide pathway, contributing to increased nitric oxide bioavailability. Nitric oxide plays an important role in regulating vascular function, mitochondrial efficiency, and muscle contractility, which has led to growing interest in nitrate-rich foods as nutritional strategies to enhance exercise performance and recovery (9). Although nitrates are not classified as polyphenols, they are frequently present in polyphenol-rich plant foods and may exert complementary physiological effects, particularly through improvements in blood flow and oxygen delivery to working muscle. Although nitrates are not classified as polyphenols, they are included in this review because many nitrate-rich foods, particularly vegetables such as beetroot and leafy greens, also contain substantial amounts of polyphenolic compounds. Moreover, nitrates and polyphenols may exert complementary physiological effects (9). While polyphenols primarily modulate oxidative stress, inflammation, and cellular signaling pathways, dietary nitrates enhance nitric oxide bioavailability, supporting vasodilation, blood flow, and oxygen delivery to working muscle. The interaction of these mechanisms may contribute synergistically to improved exercise performance, tissue perfusion, and recovery, which justifies the consideration of nitrate-containing foods within the broader nutritional strategies discussed in this review.

Cancer represents a significant global health challenge. Beyond uncontrolled cellular proliferation, it also produces extensive systemic effects on metabolic processes, immune competence, and musculoskeletal stability. Individuals who survive cancer frequently endure muscle atrophy, exhaustion, compromised functional capacity, and a reduced quality of life, phenomena that persist throughout therapeutic interventions and into survivorship, thereby highlighting the imperative for efficacious rehabilitation methodologies (10). Cancer treatments, including chemotherapy and radiotherapy, together with treatment−related inactivity, commonly result in significant impairments to skeletal muscle health and physical function. These interventions can disrupt muscle protein synthesis, increase proteolytic signaling, and promote systemic inflammation and oxidative stress, all of which contribute to muscle atrophy and loss of strength. In parallel, cancer−related fatigue, neuromuscular dysfunction, and reduced mitochondrial capacity further compromise exercise tolerance and functional performance. Prolonged periods of physical inactivity during and after treatment exacerbate these effects, accelerating declines in muscle mass, endurance, and overall physical capacity (11, 12). Collectively, these treatment− and disease−related factors underpin the high prevalence of sarcopenia, fatigue, and functional limitation observed in cancer populations, highlighting the need for targeted strategies to preserve or restore musculoskeletal function.

Physical exercise is acknowledged as a valuable intervention to alleviate these detrimental consequences: it enhances cardiorespiratory endurance, muscular strength, and overall physical functioning, concurrently diminishing treatment-induced fatigue and improving quality of life for patients afflicted with diverse malignancies, encompassing breast and prostate cancer (13). Empirical evidence supports the assertion that exercise can mitigate numerous adverse effects associated with cancer therapies and facilitate recovery when incorporated into rehabilitation frameworks. While exercise is a cornerstone of cancer rehabilitation and effectively improves physical function and quality of life, treatment-related inflammation, oxidative stress, and muscle damage may blunt adaptive responses. Polyphenols, through their anti-inflammatory, antioxidant, and anti-apoptotic properties, may help create a more favorable physiological environment for exercise adaptation and recovery, providing a rationale for examining their combined effects (14). Preliminary and emerging clinical data indicate that polyphenols such as curcumin, resveratrol, and extracts rich in flavonoids may impact tumor biology and cellular signaling pathways, potentially augmenting the adaptations induced by exercise (15). Notwithstanding these encouraging findings, the extant research has predominantly examined exercise and polyphenols independently. Consequently, increasing attention has been directed toward understanding the potential synergistic effects of polyphenol supplementation combined with structured exercise in improving musculoskeletal recovery and functional performance in individuals with cancer. Therefore, this review aims to summarize current evidence regarding the combined effects of polyphenol supplementation and exercise on musculoskeletal recovery and functional outcomes in cancer populations, with particular emphasis on the underlying molecular and physiological mechanisms.

This review first outlines the major molecular and inflammatory pathways involved in cancer-associated musculoskeletal dysfunction, with particular emphasis on Wnt/β-catenin, PI3K/Akt, and related inflammatory mediators. It then summarizes the principal classes of polyphenols and their biological relevance, followed by a discussion of the role of exercise in musculoskeletal recovery and the potential synergistic effects of combining exercise with polyphenol supplementation in cancer populations.

## 2 Dysregulation of Wnt/β-catenin and PI3K/Akt signaling and inflammatory markers in cancer: Implications for musculoskeletal recovery and rehabilitation

In the realm of oncology and musculoskeletal rehabilitation, the dysregulation of the Wnt/β-catenin and PI3K/Akt signaling cascades, coupled with the presence of elevated inflammatory biomarkers, constitutes a pivotal factor in the advancement of tumors, compromised tissue regeneration, and diminished muscular functionality. The Wnt/β-catenin signaling pathway serves as a principal modulator of cellular proliferation, differentiation, survival, and migratory behavior. In physiological conditions, it is indispensable for maintaining musculoskeletal equilibrium, facilitating the processes of bone formation and muscle-bone interaction, which collectively promote tissue repair and regeneration (16). The Wnt/β-catenin signaling pathway plays a central role in the regulation of skeletal tissue homeostasis, including bone formation, muscle–bone crosstalk, and tissue repair processes. Activation of this pathway promotes osteoblast differentiation and supports the maintenance of musculoskeletal integrity, which is critical for recovery and physical function. However, dysregulation of Wnt/β-catenin signaling has been implicated in cancer development and progression, as abnormal activation can promote tumor cell proliferation, survival, and metastasis. In addition, alterations in this pathway may impair normal regenerative processes within musculoskeletal tissues, potentially contributing to reduced recovery capacity and functional decline in individuals undergoing cancer treatment (16). In the context of malignancy, the improper activation of the Wnt/β-catenin pathway stimulates tumorigenesis by facilitating the nuclear translocation of β-catenin and initiating the transcription of genes associated with cellular proliferation, metastasis, and resistance to therapeutic interventions. Concurrently, the PI3K/Akt signaling pathway, which is typically crucial for cellular survival, growth, and metabolic processes, is found to be constitutively activated in numerous cancers, thereby fostering tumor progression and inhibiting apoptotic processes (17).

The phosphoinositide 3−kinase/protein kinase B (PI3K/Akt) signaling pathway is a critical regulator of cell survival, growth, and metabolic homeostasis. Under physiological conditions, activation of this pathway supports protein synthesis, cellular metabolism, and resistance to stress−induced apoptosis (18). However, in many cancers the PI3K/Akt pathway becomes constitutively activated, promoting uncontrolled cell proliferation, enhanced tumor survival, and resistance to apoptosis. Importantly, these signaling pathways do not act in isolation but interact closely with inflammatory mediators such as tumor necrosis factor−α (TNF−α) and the nuclear factor−κB (NF−κB) pathway. Chronic activation of these inflammatory networks contributes to systemic inflammation, muscle catabolism, and impaired tissue repair, thereby linking cancer−related inflammation with the deterioration of musculoskeletal function and reduced recovery capacity (18).

The two signaling pathways exhibit a profound interconnection: the PI3K/Akt pathway has the capacity to phosphorylate GSK3β, thereby stabilizing β-catenin and augmenting Wnt signaling, which in turn propels oncogenic gene expression (18). Furthermore, these pathways engage with inflammatory mediators such as TNF-α and NF-κB, where persistent inflammation can further activate Wnt and PI3K/Akt signaling, thereby facilitating tumor progression (19). Crucially, these molecular perturbations not only catalyze cancer advancement but also hinder musculoskeletal recuperation. For instance, the PI3K/Akt–β-catenin axis orchestrates osteoblast proliferation and differentiation during the process of bone healing, and its dysregulation compromises the regenerative potential of bone (20). From a rehabilitative standpoint, elevated levels of inflammatory markers such as TNF-α and NF-κB contribute to chronic inflammation, muscular atrophy, fibrosis, and diminished functional capacity, thereby underscoring the necessity for interventions that simultaneously address both molecular pathways and physical functionality to optimize recovery outcomes in cancer patients.

## 3 Polyphenols (anthocyanins, flavonoids, curcumin)

Polyphenols constitute a large and diverse group of naturally occurring bioactive compounds characterized by the presence of one or more aromatic rings bearing multiple hydroxyl groups. These compounds are widely distributed in plant-based foods and beverages, including fruits, vegetables, tea, coffee, cocoa, whole grains, and various medicinal plants. Structurally, polyphenols are commonly classified into several major categories according to the number of phenolic rings and the structural elements that connect them. The principal classes include flavonoids, phenolic acids, stilbenes, and lignans. Among these, flavonoids represent the most abundant and extensively studied group and are further subdivided into several subclasses such as flavonols, flavones, flavanols (or catechins), flavanones, anthocyanins, and isoflavones. Phenolic acids are typically divided into hydroxybenzoic acids and hydroxycinnamic acids, which are frequently found in fruits, coffee, and whole grains. Stilbenes, although present in smaller quantities in the human diet, have attracted considerable scientific interest due to compounds such as resveratrol, which has been widely investigated for its antioxidant and anti-inflammatory properties. Lignans, commonly present in seeds, whole grains, and certain vegetables, are another important class that can be metabolized by intestinal microbiota into biologically active enterolignans. These polyphenolic compounds exert a wide range of biological activities, including antioxidant, anti-inflammatory, and signaling-modulatory effects, which may influence cellular pathways related to oxidative stress, inflammation, mitochondrial function, and metabolic regulation. In the context of the present review, particular attention is given to polyphenol subclasses that have been most frequently investigated for their potential interactions with exercise and their possible roles in supporting musculoskeletal health and recovery in cancer populations, including flavonoids, stilbenes such as resveratrol, and curcuminoids such as curcumin. Understanding the structural diversity and classification of polyphenols provides an important foundation for interpreting their biological functions and their potential application as complementary strategies in exercise-based rehabilitation.

### 3.1 Curcumin

**Figure 1:**
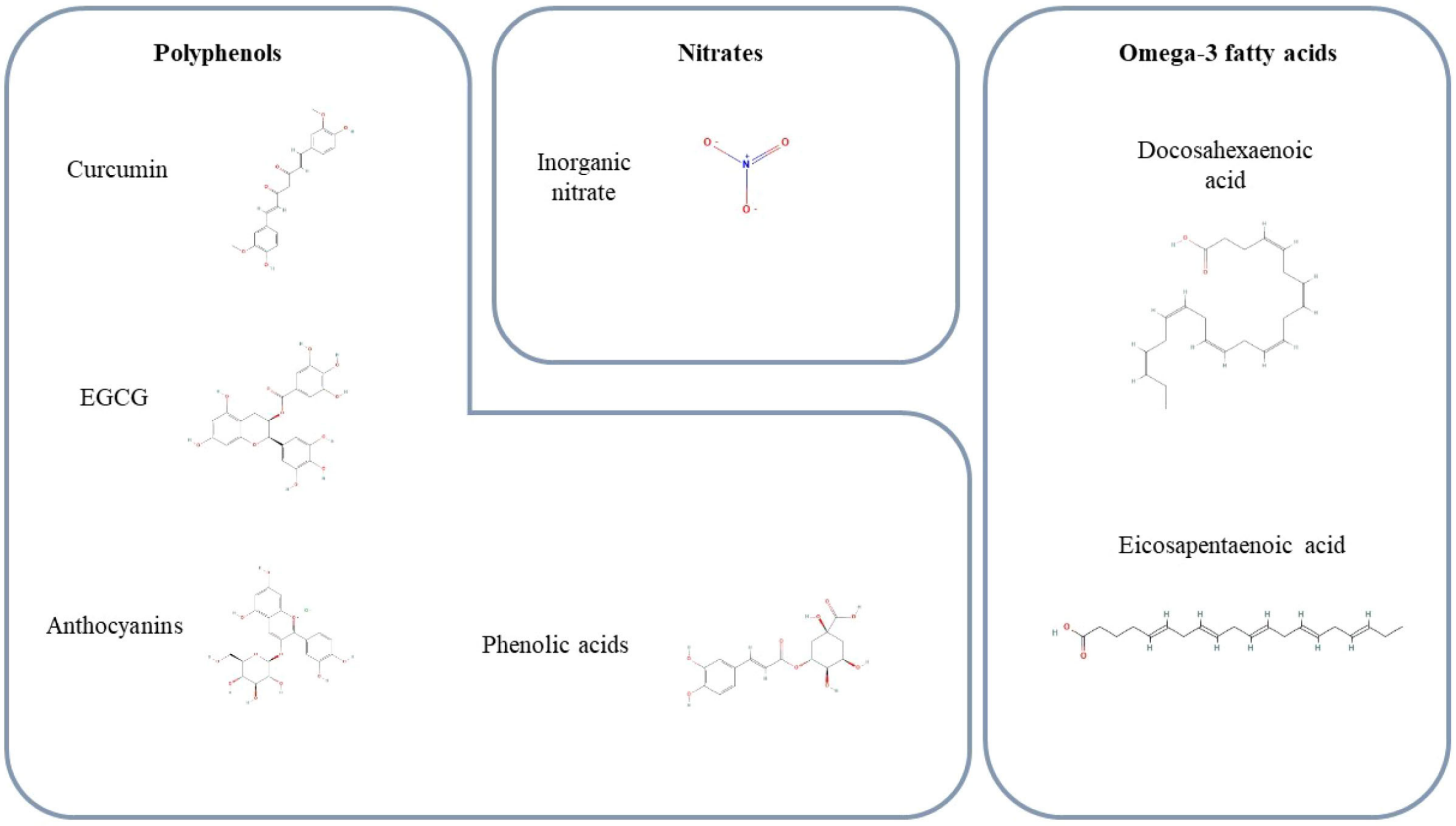
Chemical structures of key bioactive compounds discussed in the manuscript. Shown are the primary functional compounds explored for their roles in exercise performance, metabolic health, and recovery. Polyphenol structures include curcumin, epigallocatechin-3-gallate (EGCG), and the representative anthocyanin cyanidin-3-glucoside, each known for potent antioxidant and anti-inflammatory activities. The figure also illustrates inorganic nitrate (NO_3_^−^), the active molecule in nitrate-rich foods such as beetroot, spinach, and arugula, which enhances nitric oxide availability and supports vascular and muscular function. Finally, the long-chain omega-3 fatty acids eicosapentaenoic acid (EPA) and docosahexaenoic acid (DHA), derived from fish oil and algal oil—are presented for their roles in membrane fluidity, cardiovascular support, and exercise adaptations. Together, these structures summarize the major bioactive agents evaluated across the functional food categories reviewed in this manuscript.

Human trials show that curcumin can reduce soreness, inflammation, and some markers of muscle damage after strenuous exercise, particularly when delivered in enhanced-bioavailability formulations. Because native curcumin has poor absorption, multiple studies evaluated advanced delivery systems such as LipiSperse, Cureit™, and CurcuWIN^®^ (**Figure 1**). In a randomized double-blind study, 28 recreationally trained men consumed curcumin mixed with LipiSperse before and after a fatiguing lower-body resistance session. Compared with placebo, the curcumin group reported lower overall soreness at 48–72 hours, showed smaller increases in thigh circumference at 24–48 hours, and exhibited lower post-exercise lactate (7.4 vs. 8.8 mmol/L). Inflammatory markers such as IL-6, IL-10, TNF-α, and hs-CRP were also better regulated, suggesting enhanced recovery through modulation of inflammatory pathways (21). A second clinical trial evaluated Cureit™, a highly bioavailable curcumin matrix, in the context of continuous eccentric exercise meant to induce severe delayed-onset muscle soreness (DOMS). Participants taking Cureit experienced significantly reduced DOMS and modest reductions in creatine kinase, alongside slight improvements in VO_2_max, all without adverse effects. These results support the role of optimized curcumin delivery in managing inflammation and oxidative stress associated with muscle damage (22). Dose-response effects were also explored in two studies using CurcuWIN^®^. In a cohort of 63 physically active adults supplementing for eight weeks, a 200-mg curcuminoid dose preserved isokinetic knee extension torque after downhill running, while both placebo and the 50-mg dose experienced significant declines within the first 24 hours. The higher dose also prevented decreases in flexion torque and power, although soreness improvements did not reach statistical significance. A parallel report using the same study design confirmed that the lower 50-mg dose offered no meaningful advantage over placebo (23). Curcumin timing may also influence outcomes. For instance, a single-blind trial in 24 men compared 180 mg/day consumed either for seven days before eccentric arm exercise (PRE) or for four days after (POST). The POST group demonstrated greater elbow joint range of motion at days 3–4 and reduced muscle soreness at day 3 versus placebo, whereas the PRE group showed no benefits. Curcumin did not alter MVC torque or creatine kinase, indicating that its primary effects relate to soreness and stiffness rather than contractile recovery (24). Applied research in elite footballers have also shown practical translation in a sport-specific setting. Twenty-four professional players consumed a turmeric drink twice daily (60 mL each) during eight competitive matches. Compared with controls, the supplementation group showed significantly smaller increases in whole-body and leg soreness (group effects P = 0.035 and P = 0.005). CRP responses also showed a group × time interaction (P = 0.049), although CK levels and performance measures such as countermovement jump and isometric mid-thigh pull were unchanged (25). Preclinical studies align with the human data. In a mouse contusion-injury model, curcumin given daily for seven days reduced lipid peroxidation and neutrophil infiltration (lower MPO) and enhanced regeneration through increased desmin and myogenin expression, yielding outcomes comparable to diclofenac (26). Another downhill-running mouse experiment demonstrated that curcumin reduced elevations in IL-1β, IL-6, TNF-α, and serum CK at 24–48 hours while restoring treadmill run time and voluntary activity suppressed by eccentric exercise (27). Collectively, evidence from human and animal studies indicates that bioavailable forms of curcumin can reduce soreness, modulate inflammatory responses, and in some cases preserve muscle function following eccentric or high-load exercise. While effects on performance are mixed, recovery-related outcomes, pain, swelling, inflammatory cytokines, are consistently improved.

### 3.2 Tart cherry

Tart cherry is particularly rich in polyphenolic compounds, especially anthocyanins and other flavonoids, which are thought to contribute to its antioxidant and anti-inflammatory effects. These bioactive compounds may help attenuate oxidative stress, modulate inflammatory signaling, and support recovery processes relevant to exercise-induced muscle damage and cancer-associated musculoskeletal dysfunction.

Tart cherry (TC) products, particularly Montmorency varieties, have been explored extensively as nutritional aids for reducing the symptoms of exercise-induced muscle damage (EIMD). Although the overall evidence shows mixed outcomes, several well-controlled human trials suggest that TC intake may help preserve muscle function and lessen soreness following strenuous activity, depending on the dose, formulation, and exercise model used. Most acute supplementation studies have focused on short-term use surrounding a damaging bout of exercise. One trial in recreationally active adults investigated whether powdered Vistula TC taken for four days could speed recovery after 40 maximal eccentric contractions of the elbow flexors. Participants received either a spray-dried TC extract or a calorie-matched placebo. Across 72 hours of follow-up, both groups exhibited significant declines in maximal voluntary contraction, increased soreness, reduced pressure-pain thresholds, and transient swelling (all P ≤ 0.014), but none of these responses differed between groups (all P>0.3). In this context, acute powdered TC did not alter strength recovery or soreness, suggesting that very short-term dosing may not be sufficient to influence upper-arm muscle damage (28). In contrast, a 10-day regimen of a Montmorency powdered extract (480 mg/day) produced more favorable outcomes in resistance-trained men performing high-volume back squats (10×10 at 70% 1-RM). Compared with placebo, TC supplementation resulted in lower soreness ratings in both the vastus medialis and vastus lateralis, with significant differences emerging particularly at 24–48 hours’ post-exercise (vastus lateralis P = 0.024). Several blood markers showed attenuated rises in the TC group, including creatinine (P = 0.03), AST, ALT, and bilirubin, indicating reduced muscle catabolism and hepatic stress. Although inflammatory cytokines and oxidative stress markers were unaffected, lymphocyte counts normalized faster in the TC condition (P = 0.013). These findings suggest that multi-day supplementation may provide protective effects during intense lower-body loading (29).

However, not all research on lower-body muscle groups demonstrates benefits. A crossover study in recreationally active women used 1000 mg/day of concentrated TC for eight days and applied a demanding concentric–eccentric leg extension protocol. While the exercise resulted in the expected reductions in peak torque and increases in soreness over 72 hours (p < 0.001 for time), the supplement did not influence strength loss, power output, work performed, neuromuscular activation, or soreness ratings (30). Similarly, another trial using a TC juice blend reported selective benefits: after eccentric elbow flexion exercise, strength loss averaged just 4% with TC versus 22% with placebo (P<0.0001), and pain was also lower (P = 0.017), although relaxed elbow angle and tenderness were unchanged (31). Beyond laboratory settings, longer supplementation windows have been examined in athletes and individuals with chronic knee pain. Among long-distance relay runners ingesting 355 mL tart cherry juice twice daily for seven days before competition and on race day, those receiving TC reported substantially smaller increases in pain (12 ± 18 mm versus 37 ± 20 mm on a 100-mm VAS; P<0.001). Participants also expressed greater satisfaction and willingness to use the product again (32). Conversely, in recreationally active adults with patellofemoral pain, taking 60 mL/day of TC concentrate for six weeks did not improve pain scores, sleep quality, psychological wellbeing, inflammatory markers, or knee biomechanics, with placebo surprisingly showing more improvement in KOOS-PF scores (33). A recent systematic review and meta-analysis synthesized ten trials and found that TC juice improved maximal isometric strength by roughly 9% (WMD 9.13%, 95% CI: 6.42–11.84) and reduced IL-6 and IL-8 concentrations. Creatine kinase, C-reactive protein, TNF-α, and perceived soreness showed no pooled effect. A nonlinear relationship between daily TC dose and strength outcomes was also identified (34). Overall, TC supplementation shows promise for blunting strength loss and soreness after strenuous activity, particularly when taken for several days before and after exercise. However, effects vary by dose, preparation, training status, and muscle group, highlighting the need for standardized dosing strategies and larger long-term trials.

### 3.3 Quercetin

Quercetin is one of the most widely consumed dietary flavonols and has attracted considerable attention for its anti-inflammatory, antioxidant, and performance-modulating properties (35). Although the evidence remains mixed, several controlled human trials suggest that quercetin can influence post-exercise muscle function, perceived exertion, and biomarkers of inflammation, with effects dependent heavily on the dose, formulation, and training status of participants (36). Several studies have tested quercetin as an acute ergogenic aid during metabolically demanding exercise. One randomized, placebo-controlled crossover study in recreationally active adults provided 1,000 mg/day of quercetin for seven days before a cycling protocol. Quercetin supplementation led to higher absolute and relative power outputs during the incremental test and significantly reduced perceived exertion, despite no measurable changes in circulating MMP-9 or TNF-α. Interestingly, individuals who exhibited the greatest decrease in perceived exertion also showed larger reductions in CRP, hinting at inter-individual responsiveness. Another trial examining the combined use of vitamin C (160 mg/day) and quercetin (500 mg/day) during six weeks of endurance training found that while cardiovascular fitness improved in both groups, supplementation had no additive effect on heart rate, VO_2_max, body composition, or hematological indices. Together, these findings suggest that short-term high-dose quercetin may lower effort perception during demanding exercise, but chronic moderate dosing does not necessarily amplify endurance adaptations. Quercetin has also been evaluated for its ability to mitigate exercise-induced muscle damage (EIMD). A six-week resistance training program combined with 1,000 mg/day quercetin produced notable improvements in cardiorespiratory endurance, muscular strength, and power compared with placebo. Participants experienced lower post-exercise pain scores and greater gains in back and leg strength, accompanied by modest reductions in triglycerides and increases in HDL, though inflammatory markers and body composition remained unchanged. These results align with the notion that quercetin may enhance recovery-related adaptations by modulating oxidative stress and supporting training tolerance. Evidence from prolonged loading models has been less consistent. In a three-day repeated-activity protocol, quercetin did not reduce IL-6, IL-8, ICAM-1, or creatine kinase levels, nor did it affect joint-range pain following concentric, eccentric, or plyometric exercise. Similarly, in a study examining combined quercetin and cocoa flavanol supplementation over two weeks, improvements in cycling performance, ventilatory threshold, and oxygen cost were observed in both men and women; however, changes could not be attributed specifically to quercetin because the intervention used a multi-compound formulation. These results reflect a trend where quercetin’s benefits may be more pronounced in structured training programs rather than highly damaging or prolonged exercise models.

Another important factor is the delivery system. One trial comparing quercetin beverages with and without additional nutrients found no significant differences in performance or recovery outcomes. However, supplementation produced notable improvements in lipid profiles—lower LDL and triglycerides and higher HDL—independent of the co-formulated nutrients, suggesting that quercetin’s systemic metabolic effects may not always translate to acute performance changes. While human evidence centers mostly on strength recovery and endurance performance, mechanistic insight from preclinical models provides additional context. An *in vivo* and *in vitro* investigation of quercetin in tendon aging demonstrated that quercetin enhanced tendon-derived stem/progenitor cell viability, improved collagen matrix organization, and activated TSC-to-tenocyte differentiation. These findings, though preliminary, support the idea that quercetin may influence connective tissue remodeling—a potential long-term benefit for injury prevention and rehabilitation, even if not yet validated in athletic populations. Overall, research on quercetin shows promising but heterogeneous effects. Benefits appear most consistent for reducing perceived exertion, supporting strength gains during structured training, and improving lipid-related health markers. However, responses vary by dose, training background, and exercise model. Although mechanistic work suggests potential roles in tissue repair and inflammation control, larger standardized trials are needed to clarify optimal dosing strategies and to determine whether quercetin’s biological effects meaningfully translate to musculoskeletal recovery in athletes.

### 3.4 Green tea catechins

Green tea and its primary catechin, epigallocatechin gallate (EGCG), have been explored extensively for their roles in energy metabolism, oxidative stress control, and recovery from demanding exercise. Although results across studies vary, a consistent theme is that catechins tend to influence metabolic efficiency and redox balance more reliably than they alter acute performance outcomes. Work in trained cyclists has shown that EGCG alone does not mimic the well-known ergogenic effects of caffeine.

In a tightly controlled three-way crossover trial, cyclists ingested either 270 mg EGCG, a matched placebo, or placebo plus caffeine (3 mg/kg) for six days before completing a 60-min ride at 60% VO_2_max followed by a 40-km time trial. Caffeine predictably increased heart rate and plasma glucose during submaximal work, but EGCG produced no detectable changes in substrate use or time-trial performance (37). Fat and carbohydrate oxidation patterns remained virtually identical to placebo, suggesting that, at least acutely, EGCG does not meaningfully shift endurance metabolism in trained athletes (37) (**Table 1**). Findings are more encouraging under conditions of accumulated fatigue. In a 15-day triple-blind trial, amateur athletes supplemented with 500 mg/day green tea extract showed reduced indices of muscle damage and oxidative stress during repeated days of submaximal cycling (38). The placebo group exhibited impaired neuromuscular activation and higher oxidative by-products. However, the green tea–supplemented athletes-maintained muscle recruitment efficiency and had lower biochemical evidence of cellular stress. These improvements indicate that catechins may be particularly useful when athletes face day-to-day training loads that limit full recovery (38). However, not all markers respond uniformly. In soccer players, a single 640-mg dose of green tea polyphenols before an exhaustive resistance-based endurance protocol increased plasma catechin levels but did not meaningfully alter lipid peroxidation (TBARS), antioxidant enzyme activity, or CK release over 24 hours (39). Similarly, in a randomized trial involving 54 soccer athletes receiving six weeks of 450 mg/day green or sour tea extract, both teas lowered malondialdehyde (P = 0.008), yet neither influenced CK, LDH, or AST. Sour tea increased total antioxidant capacity more effectively than green tea, but neither extract improved muscle-damage indices (40). Anaerobic performance outcomes appear more sensitive to chronic supplementation combined with training. Among college students, four weeks of athletic training paired with 500 mg/day green tea extract led to significant gains in anaerobic power, mean power, and fatigue index compared with training alone. Improvements were most pronounced in the group combining supplementation with training, suggesting that catechin-related mitochondrial or antioxidant effects may interact synergistically with repeated high-intensity exercise (41).

**Table 1:** Studies investigating polyphenol roles in sport performance and muscle recovery.

Functional food/Compound/population	Study type (Human/Animal/*In Vitro*)	Population/Model	Dose & duration	Key findings	Ref
EGCG, cyclists	Human (randomized, crossover)	8 male cyclists	EGCG 270 mg (6-day period + 1 h pre-trial) vs placebo; caffeine arm 3 mg/kg	EGCG produced no meaningful benefit on fat oxidation or 60-min + 40-km performance; caffeine improved performance; HR and glucose responses noted (eg. HR: EGCG 147 ± 17 bpm).	(37)
EGCG + AA mixture (Cys/Gln/Leu)	*In vitro*/Animal/Pilot human	C2C12 cells, animal swimming model, pilot clinical trial	AA: EGCG ratio 1:1:3; animal and pilot human dosing not fully specified in abstract	*In vitro*: ↑AMPK, SIRT1, PGC-1α and ↓ROS/MDA. Animals: ↑swimming endurance, glycogen, ATP, ↓CPK/LDH. Pilot human: improved lactate clearance and higher post-exercise glucose vs placebo.	(45)
GTP, soccer players	Human (randomized, double-blind)	16 soccer players	Single acute dose GTP 640 mg prior to endurance test	Acute GTP slightly ↑plasma catechins but did **not** attenuate exercise-induced oxidative stress or muscle damage markers (TBARS, CK, SOD unchanged vs placebo).	(39)
GTE, cumulative fatigue	Human (triple-blind RCT)	16 trained males	GTE 500 mg/day for 15 days	Under cumulative-fatigue protocol, GTE reduced magnitude of muscle damage & oxidative stress and preserved neuromuscular function vs placebo — suggesting benefit in repeated-session contexts.	(38)
GTE vs sour tea, soccer players	Human (randomized, double-blind, 3 groups)	54 male soccer players	GTE 450 mg/day or sour tea 450 mg/day for 6 weeks	Both GTE and STE lowered MDA (P = 0.008); STE ↑TAC more than GTE (P = 0.01). No significant effects on muscle-damage indices (CK, AST, LDH).	(40)
Green tea extract + training (RAST)	Human (intervention)	30 college students (mixed sex)	Single 500 mg acute or 500 mg + 4 weeks training	500 mg + 4 weeks training (Group 3) improved anaerobic power, fatigue index and mean power (P<0.05), especially in females; single dose gave some female-specific improvements.	(41)
GTE in sprinters (antioxidant markers)	Human (double-blind crossover)	16 sprinters	GTE providing 980 mg polyphenols/day for 4 weeks	GTE ↑total polyphenols & TAC at rest, ↓MDA and SOD after sprint test; no change in sprint performance vs placebo, antioxidant effect without ergogenic benefit.	(42)
dGTE, recreational males	Human (parallel RCT)	14 recreational males	dGTE 571 mg/day for 4 weeks	dGTE ↑fat oxidation by 24.9% (0.241→0.301 g·min^−^¹; P = 0.05), ↓body fat by 1.63% (P<0.001) and improved distance covered by 10.9% (20.23→22.43 km; P<0.001).	(43)
Tannase-treated green tea, older adults	Human (12-week RCT)	Older adults (frailty context)	600 mg/day for 12 weeks	↑isokinetic flexor strength and handgrip vs placebo; prevented arm muscle mass loss; ↓myostatin correlated with muscle changes, suggests benefit for age-related muscle decline.	(44)
Quercetin, neuromuscular protection	Human (randomized, crossover)	12 young men	Quercetin 1000 mg/day for 14 days	Attenuated strength loss after severe eccentric exercise: ↑MVIC (+4.7% vs baseline), reduced torque and MFCV decay vs placebo, suggests protection vs EEIMD.	(97)
Quercetin, eccentric contractions (24)	Human (double-blind lab)	30 healthy subjects	1000 mg/day (7 d pre and 5 d post)	Plasma quercetin reached 202 ± 52 ng/mL; large exercise-induced damage observed but **no** treatment effect on strength loss, soreness, CK, IL-6 or CRP vs placebo.	(98)
Quercetin 8-week trial, students	Human (double-blind)	60 male students	Quercetin 500 mg/day ± vit C for 8 weeks	↑lean body mass, total body water, BMR, TEE in quercetin groups; VO2max rose non-significantly — some performance indices improved.	(99)
Quercetin in badminton players	Human (8-week RCT)	26 badminton players	Quercetin 1000 mg/day for 8 weeks	No change in VO2max, lactate or body fat; TTE (time-to-exhaustion) increased in quercetin group (P<0.05) — suggests endurance benefit without other changes.	(100)
Quercetin, IGF and recovery	Human (crossover)	12 young men	Quercetin 1 g/day for 14 days	Faster and greater IGF-I/II response during recovery; significant reductions in CK (p<0.005), LDH (p<0.001), Mb (p<0.05) and IL-6 (p<0.05) vs placebo — indicates improved recovery biology.	(101)
Acute quercetin, resistance session	Human (acute crossover)	10 young men	Single dose 1 g, 3 h pre-exercise	Improved torque-velocity curve, smaller MVIC reduction (0.91 ± 6.10% vs 8.66 ± 5.08%), +10.6% rate of torque development, +28.2% neuromuscular efficiency; greater exercise volume (p<0.05); no change in blood markers.	(102)
Quercetin, tendon stem cells (mechanistic + delivery)	*In Vitro*/Animal	Senescent TSPCs; aged rat Achilles model	Quercetin treatment *in vitro*; DPH@QUE local delivery *in vivo*	Quercetin reduced SASP, restored mitochondrial function (AKT pathway modulation); DPH@QUE hydrogel delivered anti-senescent and reparative effects in aged tendon model.	(103)
Quercetin, 7-day/14-day recovery (EEIMD)	Human (double-blind crossover)	16 men	1000 mg/day for 14 days	Q attenuated strength loss and improved recovery of neuromuscular function and biochemical markers across 7 days post-EEIMD (especially at higher angular velocities; p<0.02).	(104)
Tart cherry, powdered Vistula (pilot)	Human (pilot, crossover)	22 recreationally active	Powdered TC vs placebo, 4 days starting day of exercise	No accelerated recovery vs placebo across MVC, soreness, PPT, ROM, girth (p>0.3) despite time effects, acute powdered Vistula TC not effective in this protocol.	(28)
Tart cherry, CherryPURE resistance	Human (double-blind)	23 resistance-trained men	480 mg/day for 10 days (incl. day of exercise → 48 h post)	↓muscle soreness in vastus medialis/lateralis (some p ≤ 0.024), ↓markers of catabolism (AST, creatinine, bilirubin, ALT at various times); no changes in classic inflammatory markers.	[TC-2]
Tart cherry, concentrated, women	Human (cross-over)	17 recreational women	1000 mg/day for 8 days (exercise on day 4)	No effect on isokinetic peak torque, peak power, total work or muscle soreness vs placebo; time effects only.	(30)
Tart cherry juice blend, eccentric elbow flexion	Human (crossover RCT)	14 male college students	12 fl oz twice daily for 8 days	Strength loss averaged 22% (placebo) vs 4% (cherry juice) over 4 days post-eccentric; pain also significantly reduced (strength p<0.0001; pain p=0.017).	(31)
Tart cherry, patellofemoral pain trial	Human (6-week RCT)	24 recreational adults with PFP	Montmorency concentrate 60 mL/day for 6 weeks	No benefit vs placebo on KOOS-PF, biomarkers or biomechanics; placebo group had some KOOS-PF improvement.	(33)
Tart cherry, endurance relay runners	Human (field RCT)	54 runners (36M/18F)	355 mL twice daily for 7 days prior + race day	Cherry juice group reported smaller pain increase post-race (12 ± 18 mm) vs placebo (37 ± 20 mm), p<0.001, practical pain reduction in ultra/long-distance setting.	(32)
Curcumin + LipiSperse, resistance exercise	Human (randomized, double-blind)	28 recreational males	Curcumin (LipiSperse form) drink pre & post; assessed up to 72 h	Lower postexercise capillary lactate (7.4 vs 8.8 mmol/L), less perceived pain at 48–72 h, reduced thigh circumference at 24–48 h, suggests faster recovery/less edema.	(21)
Cureit™ bioavailable curcumin, DOMS	Human (double-blind RCT)	Participants with continuous eccentric exercise protocol	Cureit™ oral (dose not specified in abstract)	Significant reduction in DOMS vs placebo, slight ↓CK and slight ↑VO2max; safe and well tolerated — highlights benefit of bioavailable formulations.	(22)
CurcuWIN^®^ dose trial, downhill running	Human (8-week RCT)	63 active men & women	CurcuWIN^®^ 250 mg (50 mg curcuminoids) or 1000 mg (200 mg curcuminoids) for 8 weeks	200-mg curcuminoids (1000 mg product) attenuated some performance decrements after downhill run vs placebo; 50-mg dose offered no benefit. Soreness increased in all groups; some non-significant trends favor higher dose.	(23)
Curcumin timing study	Human (single-blind)	24 young men	180 mg/day: PRE group 7 d before or POST group starting 4 d after; CON placebo	POST ingestion lowered muscle soreness at day 3 and improved ROM at days 3–4 vs CON; PRE group showed no benefit — suggests post-exercise timing may be better.	(24)
Curcumin, contusion injury in mice	Animal (mouse)	Mass-drop contusion gastrocnemius model	Oral curcumin once daily for 7 days post-injury (dose not in abstract)	↓lipid peroxidation & MPO, ↑desmin (satellite cell regeneration) and modulation of IKK-α/β/myogenin, curcumin accelerated muscle repair vs diclofenac and untreated.	(26)
Curcumin, downhill running mice	Animal (mouse)	Trained male mice; downhill/uphill treadmill runs	Curcumin feeding (duration not in abstract) around runs	Curcumin blunted increases in IL-1β, IL-6, TNF-α and CK after downhill running and preserved treadmill run-to-fatigue & voluntary activity, supports anti-inflammatory and performance-protective effects *in vivo*.	(27)
Turmeric drink, elite footballers	Human (applied field study)	24 elite male footballers	Turmeric drink 60 mL twice daily across eight matches (timing per study)	Group × time effect for CRP and smaller increases in subjective soreness (p values 0.005–0.035); no effects on CK, CMJ or IMTP, suggests reduced inflammation/soreness in applied setting.	(25)

Research in sprint athletes adds further nuance. In a four-week crossover trial using 980 mg/day of green tea polyphenols, sprinters showed elevated resting antioxidant capacity and reduced post-test oxidative markers; specifically, lower malondialdehyde and superoxide dismutase activity after repeated sprint testing. Nevertheless, sprint performance remained unchanged, and no reductions in CK were observed. These results highlight that EGCG bolsters antioxidant defenses without necessarily altering high-intensity output (42). Decaffeinated extracts have also shown metabolic benefits. Four weeks of 571 mg/day decaffeinated green tea extract in recreationally active men increased fat oxidation by ~25%, reduced body fat by approximately 1.6%, and improved cycling performance distance by around 11% compared with placebo. These adaptations occurred without major shifts in free fatty acids or blood pressure, supporting a direct catechin-mediated enhancement of substrate use (43). Finally, a 12-week study in older adults found that tannase-treated green tea extract (600 mg/day), a form enriched in epicatechin and gallic acid, helped preserve arm muscle mass and significantly increased handgrip and isokinetic flexor strength. Notably, the intervention reduced circulating myostatin, suggesting a potential pathway through which catechins support age-related muscle maintenance even in the absence of structured exercise (44). Mechanistic research provides a strong rationale for these human findings. EGCG combined with amino acids (cysteine, glutamine, leucine) enhanced mitochondrial biogenesis markers (AMPK, SIRT1, PGC-1α) and lowered reactive oxygen species in C2C12 myoblasts. In rodents, the same mixture raised muscle ATP and glycogen stores, reduced fatigue-related enzymes, upregulated NRF2 pathways, and significantly increased endurance capacity. A pilot human arm of the study reported faster lactate clearance after exercise (45).

## 4 Nitrates (beetroot, spinach, arugula)

Although beetroot juice is widely studied for its high nitrate content and its role in enhancing nitric oxide bioavailability, it also contains a variety of bioactive phytochemicals, including polyphenols and betalains, which possess antioxidant and anti−inflammatory properties. The inclusion of beetroot within this review reflects the broader concept that whole plant foods often provide multiple classes of bioactive compounds that may act through complementary mechanisms. In this context, nitrates may primarily influence vascular function, oxygen delivery, and muscle efficiency via nitric oxide production, whereas polyphenols contribute to the modulation of oxidative stress, inflammation, and cellular signaling pathways associated with exercise-induced muscle damage and recovery. Considering these overlapping and potentially synergistic mechanisms helps situate beetroot-based interventions within the broader framework of nutritional strategies targeting musculoskeletal function in cancer populations.

### 4.1 Beetroot

Beetroot juice (BRJ) has been extensively evaluated in human exercise settings, with numerous trials examining its role in muscle recovery, neuromuscular function, and performance under both acute and short-term supplementation protocols. Although mechanisms are often attributed to dietary nitrate and its downstream nitric oxide (NO)–mediated effects, many BRJ products also contain antioxidants and polyphenols that may contribute to recovery. The studies summarized below focus exclusively on human research, as no relevant *in vivo* or *in vitro* work was provided. A growing line of work suggests that beetroot supplementation may help reduce symptoms associated with exercise-induced muscle damage (EIMD). In young female volleyball athletes, two days of repeated BRJ dosing (eight 50-mL servings) following an EIMD protocol improved several markers of recovery compared with placebo. Athletes demonstrated better wall-sit endurance and experienced less thigh swelling and reduced muscle soreness up to 48 hours’ post-exercise (P<0.05), although vertical jump height and sit-and-reach performance were unaffected. These findings indicate that BRJ may offer early benefits for localized muscle endurance and discomfort in female populations, an area historically underrepresented in supplementation trials (46). Similar effects have been reported in male team-sport athletes. In a study using two repeated-sprint tests 72 hours apart, three days of beetroot juice (2×250 mL/day) helped preserve countermovement jump (CMJ) height and reactive strength index (RSI). At 72 hours’ post-test, CMJ and RSI were notably higher with BRJ than placebo (7.6% and 13.8% greater, respectively; P<0.05). Pressure-pain threshold was also elevated 24 hours after the second sprint test. Despite these neuromuscular benefits, sprint performance, maximal voluntary contraction, creatine kinase, and oxidative stress markers were not altered, showing that BRJ may improve functional recovery without necessarily modifying biochemical indices (47). Acute supplementation may also benefit athletes exercising in demanding environments. In a trial involving 27 climbers who consumed 70 mL of BRJ (≈400 mg nitrate) before ascending to 3720 meters, the BRJ group demonstrated superior lower-body strength, handgrip force, flexibility, horizontal jump performance, and estimated VO_2_max compared with both placebo and water controls. Muscle soreness in the gastrocnemius was significantly lower 24 hours after descent (P = 0.003), though no changes were observed for the quadriceps or hamstrings. These results suggest that BRJ can support neuromuscular resilience even in hypoxic conditions (48).

Dose-dependent effects have also been explored. When recreationally active men consumed either high-dose (250 mL) or low-dose (125 mL) BRJ after eccentric exercise, both regimens improved pressure-pain threshold across 24–72 hours and accelerated the recovery of CMJ performance, particularly in the high-dose group. However, inflammatory cytokines (IL-6, IL-8, TNF-α) and CK were not altered by either dosage, highlighting once again that BRJ’s benefits may act primarily through functional rather than biochemical pathways (49). Beetroot supplementation has also been tested in resistance training. In resistance-trained men, three days of BRJ providing 450 mg nitrate/day increased repetitions to failure at 80% 1RM in both bench press and back squat, improved movement velocity, and enhanced muscle oxygenation (SmO_2_). Cardiovascular strain was reduced, with lower peak heart rates and better heart-rate variability recovery. Muscle soreness in the upper body was also lower in the following 24–48 hours (50). Not all studies report benefits. In experienced marathon runners, three days of post-race BRJ did not reduce soreness, inflammation, or muscle damage markers compared with placebo, likely because these athletes displayed relatively small post-race elevations in damage indices (51). Finally, chronic loading may help intermittent sports. Six days of BRJ (~800 mg nitrate/day) improved Yo-Yo IR1 performance by 3.4% in trained soccer players, accompanied by reduced heart rate and elevated plasma nitrate/nitrite levels (52). Overall, human evidence shows that beetroot juice consistently improves neuromuscular recovery, functional performance, and perceived soreness across various exercise modalities, although effects on biochemical markers remain inconsistent.

### 4.2 Nitrate salts and concentrates

**Figure 2:**
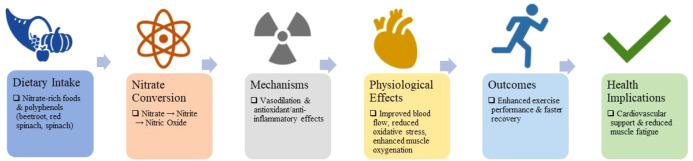
Graphical summary of the effects of dietary nitrate- and polyphenol-rich foods on exercise performance and recovery. Dietary intake of nitrate-rich foods such as beetroot, red spinach, and spinach increases nitrate and nitrite levels, promoting nitric oxide production. This enhances vasodilation, antioxidant, and anti-inflammatory responses, which improve muscle oxygenation, reduce oxidative stress, and support cardiovascular function. Consequently, these mechanisms contribute to enhanced exercise performance, faster recovery, and reduced muscle fatigue.

Research using purified nitrate sources, such as sodium nitrate or nitrate-enriched beverages stripped of other beetroot compounds, offers a clearer picture of how nitrate alone influences exercise physiology (**Figure 2**). Most existing evidence in this category comes from human trials, and the overall pattern is that nitrate reliably boosts circulating NO-related metabolites but does not consistently improve performance, particularly in highly trained athletes. Studies using sodium nitrate loading demonstrate strong biochemical responses without parallel improvements in endurance. When trained cyclists consumed sodium nitrate for three days (10 mg/kg/day), plasma nitrate increased more than fivefold and nitrite nearly doubled. Despite this, athletes covered virtually the same distance during a 40-minute cycling test (26.4 vs. 26.3 km), with no meaningful differences in power output. Notably, endothelin-1 rose more sharply after exercise with nitrate, while nitrated protein levels remained unchanged, suggesting limited downstream physiological impact (53). Acute sodium nitrate ingestion (10 mg/kg, taken three hours before testing) produced similarly large increases in nitrate (from ~29 to 250 µM) and nitrite (from ~1998 to 2313 nM). Although VO_2_peak decreased modestly (4.82 to 4.64 L/min; P = 0.01), indicating improved oxygen efficiency, athletes did not achieve greater maximal power or longer exhaustion times. Thus, isolated nitrate appears to make exercise more economical without translating these changes into better performance (54). When nitrate-rich beverages designed to mimic a pure nitrate effect were tested in elite cyclists, results remained largely neutral. Six days of supplementation raised plasma NOx substantially but had no effect on VO_2_ kinetics, cycling economy, time-trial outcomes, or repeated-sprint power (55). A larger trial in 26 national-level cyclists also found that nitrate consumed 75–150 minutes before two 4-minute time trials did not enhance performance (56). In the second trial, nitrate might even have impaired outcomes slightly. More favorable results emerge in moderately trained cyclists. After drinking 0.5 L of nitrate-rich beetroot juice (~6.2 mmol) 2.5 hours before testing, club-level cyclists improved their 4-km time by 2.8% and their 16.1-km time by 2.7%. These gains were driven by higher sustained power output (292 vs. 279 W for 4 km; 247 vs. 233 W for 16.1 km), despite no differences in VO_2_. These findings reflect a population that may be more responsive to nitrate-based efficiency improvements (57). Short-term loading protocols lasting 6–8 days show mixed outcomes. In one study, cyclists consuming ~4 mmol of nitrate daily for eight days posted likely meaningful improvements in 4-km time-trial performance (0.7% faster) and generated ~2.4% more power, even though VO_2_peak and ventilatory thresholds were largely unchanged (58). However, when highly trained cyclists followed a 3–7-day dosing regimen with ~8 mmol/day, neither 1-km nor 4-km time-trial results improved. After four days of supplementation, performance in the 1-km event was even classified as “likely harmful” (59). Beyond cycling, nitrate concentrates have been tested in swimming and hypoxic exercise. In trained swimmers completing a maximal 168-m backstroke time trial, nitrate (~12.5 mmol) elevated exhaled NO markedly but produced trivial overall performance changes. A minor benefit appeared only in the latter half of the trial (60). Under simulated high-altitude conditions (4300 m), a 15-mmol sodium nitrate dose raised plasma NOx tenfold and lowered mean arterial pressure, yet forearm blood flow, muscle oxygenation, and grip endurance remained unchanged (61). Taken together, nitrate salts and isolated nitrate concentrates consistently stimulate nitric oxide pathways but deliver inconsistent performance benefits. Improvements appear most likely in moderately trained athletes during prolonged time trials, while elite performers and short-duration high-intensity tasks show minimal responsiveness.

### 4.3 Spinach and arugula

Leafy-green vegetables such as spinach and arugula are among the richest natural sources of dietary nitrate, supporting nitric oxide (NO) production through the nitrate-nitrite-NO pathway. Although beetroot has received the most attention, several recent controlled trials have evaluated spinach-derived nitrate and red spinach extract as functional ingredients for vascular modulation, fatigue resistance, and exercise performance. Overall, evidence suggests that spinach-based nitrate intake can enhance NO bioavailability, lower blood pressure, and improve performance in select exercise modalities, although effects vary across populations and exercise types. Multiple studies have evaluated RSE, a concentrated spinach extract standardized for nitrate content, across endurance, sprint, and resistance-training contexts. One randomized, double-blind, cross-over trial in recreationally active young adults (n=17) demonstrated that seven days of RSE (1 g/day, taken daily and again one hour before exercise) improved several performance metrics during a 4-km cycling time trial compared with placebo. Time to completion decreased from 410.6 ± 31.3 s to 404.6 ± 24.6 s (P = 0.022), accompanied by higher average speed (35.7 ± 2.2 vs. 35.3 ± 2.5 km/h) and modest increases in absolute and relative power output (P ≤ 0.022). Notably, the performance benefit was driven primarily by female participants, who showed significant improvements across all measures. Post-exercise diastolic blood pressure was also lower with RSE (66.1 ± 6.1 vs. 70.1 ± 5.0 mmHg, P = 0.017), suggesting favorable vascular responses without altering perceived exertion or fatigue (62) (**Table 2**). A second trial focused on repeated anaerobic work in trained female collegiate athletes (n=11). Participants ingested ~400 mg nitrate from RSE or placebo two hours before completing three 15-s Wingate tests. Although RSE did not significantly change mean or peak power (P≥0.067), nor did it alter heart rate or perceived exertion, it did reduce fatigue index (P = 0.018) and produced higher post-exercise blood lactate concentrations (p = 0.030) (63). These findings imply improved lactate/H^+^ clearance rather than enhanced power output, indicating a potential role for RSE in mitigating metabolic fatigue during high-intensity intermittent efforts. Another investigation evaluated chronic RSE supplementation (4 g/day, ~6 mmol nitrate) over 15 days in healthy men (n=11). Compared with placebo, RSE lowered resting systolic blood pressure (124 ± 3 vs. 129 ± 4 mmHg, p < 0.05) and nearly doubled fractional exhaled NO (41 ± 14 vs. 21 ± 6 ppb, P<0.001). High-intensity exercise tolerance also improved, with time-to-exhaustion increasing from 642 ± 198 s to 764 ± 221 s (P = 0.024). These results support sustained nitrate intake from spinach as a viable strategy for improving NO-mediated vascular function and endurance performance (64). Resistance-training outcomes appear less responsive. In resistance-trained males (n=10), seven days of RSE providing 180 mg nitrate/day did not enhance bench press repetition performance, peak or mean power, muscle oxygenation, or cognitive function during exercise. This aligns with other nitrate research showing limited effects in well-trained strength athletes or in exercises with brief contraction phases (65).

**Table 2:** Studies investigating nitrate roles in sport performance and muscle recovery.

Population	Supplement & dose	Duration	Exercise test	Main outcomes	Ref
17 recreationally active adults (9 men, 8 women)	1 g/day RSE for 7 days + 1 g 1 h pre-test	7 days	4-km cycling TT	↓ DBP post-exercise; improved TT time, avg power, relative power, and speed. Women showed stronger response.	(62)
11 NCAA D-I female athletes	~400 mg nitrate from RSE (vs tomato juice placebo)	Acute (2 h before)	3 × 15-s Wingate	No improvement in mean/peak power; ↑ post-exercise lactate; ↓ fatigue index (better fatigue resistance).	(63)
10 resistance-trained males	2 g/day RSE (180 mg nitrate)	7 days	5 sets bench press to failure	No difference in reps, power, muscle oxygenation, or cognition.	(65)
11 healthy males	4 g/day RSE (~6 mmol nitrate)	15 days	Time-to-exhaustion test	↓ SBP; ↑ exhaled NO; ↑ time-to-exhaustion vs placebo.	(64)
13 trained athletes	NaNO_3_ 10 mg/kg/day	3 days	40-min cycling TT	↑ plasma nitrate/nitrite; no improvement in distance or power; ↑ ET-1 response.	(53)
11 cyclists	Single dose NaNO_3_ 10 mg/kg, 3 h before test	Acute	Submax + incremental test to exhaustion	↓ VO_2_ at peak and at given power; no change in time-to-exhaustion or maximal power.	(54)
10 elite cyclists	0.5 L BR/day	6 days	VO_2_ kinetics, 120-min preload + TT, repeated sprints	↑ plasma NOx; no improvements in economy, TT, or sprint performance.	(55)
26 national-level cyclists	70 mL BR taken at 75 or 150 min pre-exercise; with/without top-up	Acute	Two 4-min TTs	Effects unclear; second TT possibly harmed performance; inconsistent individual responses.	(55)
9 competitive male cyclists	0.5 L BR (~6.2 mmol nitrate) 2.5 h pre-TT	Acute	4-km and 16.1-km TTs	↑ mean power; improved 4-km (+2.8%) and 16.1-km (+2.7%) TT times.	(57)
8 competitive cyclists	70 mL BR (~4 mmol nitrate) daily	8 days	Ramp test, economy, 4-km TT	TT time likely improved (-0.7%); small unclear effects on VO2peak/economy.	(58)
9 highly trained cyclists	140 mL BR (~8 mmol nitrate) daily	3–7 days	1-km and 4-km TTs	3–4 days: likely harmful for 1-km, unclear for 4-km. 6–7 days: still no clear benefit.	(59)

Beyond nitrate effects, whole spinach may influence redox and inflammatory responses to repeated strenuous activity. In a controlled, within-participants trial, trained men (n=15) consumed 70 g/day raw spinach, a superfood blend, or no supplement for seven days before performing three 30-s Wingate tests. Both spinach and the superfood increased superoxide dismutase levels post-exercise (P = 0.035 and P = 0.010, respectively) and reduced malondialdehyde (P = 0.001 and P = 0.017) and interleukin-6 (P ≤ 0.003) (66). These data suggest that increasing dietary nitrate and phytonutrient intake through whole spinach confers measurable anti-inflammatory and antioxidant benefits, potentially supporting recovery between repeated high-intensity bouts. A randomized crossover study in healthy adults (n=30) compared one week of beetroot juice to one week of nitrate-rich vegetables, primarily spinach and arugula, providing ~400 mg nitrate/day. Both interventions elevated plasma nitrate and nitrite, with beetroot producing slightly higher concentrations (day 1 nitrate: 2.31 ± 0.56 mg/dL vs. 1.71 ± 0.83 mg/dL, P<0.001). Despite these biochemical differences, systolic and diastolic blood pressure measured 2.5 hours after lunch decreased similarly in both conditions (p < 0.05), indicating that spinach and arugula are effective whole-food alternatives to concentrated nitrate supplements (66).

## 5 Effects of exercise synergy with polyphenols on Wnt/β-catenin and PI3K/Akt signaling pathways and inflammatory markers in muscle regeneration and cancer rehabilitation

The integrated utilization of physical exercise alongside polyphenol supplementation appears to influence fundamental intracellular signaling pathways including the Wnt/β-catenin and PI3K/Akt cascades, which are crucially implicated in both muscle regeneration and outcomes related to cancer rehabilitation. While empirical clinical evidence in survivors of cancer remains sparse, preclinical mechanistic investigations suggest that this synergistic approach may enhance molecular signaling that governs tissue repair, metabolic adaptation, and anti-inflammatory responses. Exercise is recognized to exert a significant influence on muscle repair by modulating established signaling networks. The mechanical contraction and the resultant physiological stimuli activate elements of the Wnt/β-catenin signaling cascade, a pathway that has been extensively associated with the activation of satellite cells, myogenic differentiation, and the repair of adult muscle following injury or overload (for instance, satellite cell-mediated myogenesis in models of muscle regeneration) (67). Concurrently, exercise promotes the activation of the PI3K/Akt signaling pathway through both systemic and mechanotransductive signals, which enhances protein synthesis, supports cellular survival, and inhibits catabolic processes within skeletal muscle, thereby facilitating hypertrophic responses and recovery (68).

Polyphenolic compounds, including resveratrol, quercetin, and curcumin, have been shown to exert an impact on signaling networks via context-dependent modulation. In oncological models, resveratrol has been found to interact with the PI3K/Akt pathway, frequently functioning to inhibit the aberrant PI3K/Akt signaling linked to tumor cell proliferation, while concurrently engaging in antioxidant and apoptotic mechanisms that may indirectly enhance tissue resilience (69). Furthermore, polyphenols possess the ability to modulate the activity of the Wnt/β-catenin pathway; however, a significant portion of this evidence originates from non-muscle or neurodegenerative contexts. Nevertheless, this underscores the potential of bioactive plant-derived compounds to modify canonical Wnt outputs that are pertinent to cellular differentiation and repair (70). Significantly, recent mechanistic investigations utilizing integrated exercise and polyphenol interventions within animal models indicate that this synergistic approach can activate various signaling pathways, including Wnt and PI3K/Akt, relevant to cancer progression and systemic adaptation. For example, a contemporary multi-omics analysis conducted in a murine model of breast cancer demonstrated that the conjunction of curcumin supplementation and swimming exercise yielded distinct gene expression profiles that were markedly enriched in Wnt signaling and PI3K/Akt signaling, among other pathways, when contrasted with singular interventions. These collective molecular alterations correlated with enhanced anti-tumor effects at both the transcriptomic and metabolomic levels compared to either exercise or polyphenol treatment alone, implying a synergistic interaction *in vivo* (71).

Mechanistically, it has been posited that exercise-induced mechanotransduction and the concomitant metabolic signals serve to prepare muscle tissue for regeneration through the enhancement of satellite cell responsiveness (which is associated with Wnt signaling) and the amplification of anabolic signaling via the PI3K/Akt pathway. Polyphenols may augment or facilitate these adaptive responses by alleviating oxidative stress and inflammatory signaling, which can otherwise diminish anabolic signals, as well as by modulating intracellular kinase activity to promote repair and resilience in both muscular and neoplastic tissues. Furthermore, both exercise and polyphenols converge upon shared intracellular nodes such as AMPK and downstream effectors that intersect with the PI3K/Akt pathway and related growth signaling cascades, thereby further emphasizing their potential synergistic effects on cellular homeostatic processes pertinent to rehabilitation and functional recovery (72). In aggregate, although empirical investigations involving human subjects remain limited, the insights derived from mechanistic studies suggest that physical exercise collaborates with polyphenol supplementation to modulate essential signaling pathways specifically, Wnt/β-catenin and PI3K/Akt, thereby enhancing muscle regeneration and providing promising molecular foundations for improved rehabilitation strategies in individuals who have survived cancer. Subsequent research endeavors should concentrate on elucidating the precise activations of these pathways within clinical cohorts and clarifying parameters such as dosage, timing, and types of exercise to maximize the efficacy of these molecular interactions.

## 6 Cancer-specific preclinical and clinical evidence

Although physical activity and dietary polyphenols have each exhibited advantageous influences on oncological outcomes and musculoskeletal health, there exists a paucity of direct evidence from integrated interventions within cancer models. This segment encapsulates the principal preclinical and clinical discoveries across prominent cancer types and underscores the significant deficiency in randomized controlled trials assessing the synergistic effects of exercise in conjunction with polyphenols in cancer rehabilitation.

### 6.1 Breast cancer

Emerging research suggests that combining exercise and polyphenol intake could synergistically influence tumor biology and functional outcomes in breast cancer models. The research examined the synergistic effects of curcumin supplementation in conjunction with swimming exercise on breast cancer utilizing a murine model. In comparison to individual treatment modalities, the combination demonstrated significantly enhanced anti-tumor efficacy, as substantiated by reductions in tumor size, *in vivo* imaging assessments, and comprehensive histopathological evaluations. Multi-omics investigations disclosed 445 differentially expressed genes (154 exhibiting upregulation, 291 displaying downregulation) alongside pronounced metabolic alterations. Functional enrichment analyses indicated that the calcium, Wnt, PI3K/Akt, and IL-17 signaling pathways are pivotal in mediating the observed anti-cancer effects, whereas metabolomic evaluations underscored modifications in amino sugar/nucleotide sugar metabolism and amino acid biosynthesis. O2PLS analytical approaches identified the most prominent genes and metabolites that exhibit the strongest association with these therapeutic effects. Collectively, the investigation offers mechanistic insights at both transcriptomic and metabolomic strata regarding the synergistic interplay between curcumin and exercise in combating breast cancer (71) (**Table 3**). Another study meticulously examined the ramifications of an eight-week regimen of high-intensity interval training (HIIT) in conjunction with curcumin supplementation on cardiac signaling pathways in murine models subjected to doxorubicin treatment for cancer. Doxorubicin administration resulted in cardiac impairment characterized by heightened levels of ERK1/2 and IL-18, alongside diminished PI3K concentrations. The integrative approach of exercise combined with curcumin supplementation significantly attenuated the levels of ERK1/2 and IL-18, while concurrently restoring PI3K levels in comparison to the application of doxorubicin alone. Although both interventions exhibited individual beneficial effects, their synergistic application yielded markedly enhanced cardioprotective outcomes, thereby underscoring a prospective synergistic role in alleviating chemotherapy-induced cardiac damage (73). One study assessed the synergistic effects of HIIT in conjunction with quercetin supplementation on tumor angiogenesis within a breast cancer murine model. Mice that were subjected to the combined regimen of HIIT and quercetin (THQ group) exhibited a marked decrease in the expression levels of pivotal angiogenic genes TIE-2 and VEGF-A in comparison to both the tumor-only and HIIT-only cohorts. These findings imply that physical exercise may work in concert with quercetin to impede tumor angiogenesis, thereby illuminating a prospective molecular mechanism through which lifestyle modifications could enhance cancer therapeutic strategies (74).

**Table 3:** Effects of exercise synergy with polyphenols on Wnt/β-catenin and PI3K/Akt signaling pathways and inflammatory markers in muscle regeneration and cancer rehabilitation.

Subjects/Model	Intervention	Duration/Dosage	Key findings/Molecular effects	Ref
Breast cancer mice	Curcumin treatment + swimming exercise	Not specified	- 445 differentially expressed genes (154 ↑, 291 ↓) - Key pathways: calcium signalling, Wnt, PI3K/Akt, IL-17 - Differential metabolites: amino sugar & nucleotide sugar metabolism (chitosan, D-glucosamine 6-phosphate, L-fucose, N-acetyl beta-mannosamine); amino acid biosynthesis (DL-isoleucine, DL-tyrosine, homocysteine) - O2PLS revealed top-ranked genes and metabolites	(71)
Rats with doxorubicin-treated breast cancer	HIIT + curcumin (100 mg/kg)	8 weeks; HIIT: 6 × 3 min 20 sec intervals at 80–95% VO_2_max, 2 min active recovery	- ERK1/2 ↑ in cancer + doxorubicin; ↓ with HIIT + curcumin - PI3K ↓ in cancer + doxorubicin; ↑ with HIIT + curcumin - IL-18 ↑ in cancer + doxorubicin; ↓ with HIIT + curcumin	(73)
Female Balb/C mice with estrogen receptor-dependent breast cancer	HIIT + quercetin (110 mg/kg)	6 weeks; treadmill 1 h/session, 3 days/week	- THQ group showed significant ↓ in TIE-2 and VEGF-A expression vs. T group - Quercetin further ↓ TIE-2 and VEGF-A vs. HIIT alone	(74)
Female BALB/C mice with 4T1 breast cancer	Endurance exercise (5 days/week) + curcumin gavage (6×/week)	5 weeks	- Significant ↓ in tumor growth, TNF-α and NF-κB expression in EC group vs. E, CC, C - EC more effective than single interventions	(75)
Female BALB/C mice with 4T1 breast cancer	Endurance exercise (5 days/week) + curcumin gavage (6×/week)	5 weeks	- Tumor growth ↓ in all interventions vs. control - EC significantly ↓ intratumoral IL-4 & STAT6 expression vs. control - Exercise alone ↓ both genes; curcumin alone ↓ STAT6	(76)
Female BALB/C mice; *in vitro* U937 macrophage model	Genistein + moderate-intensity exercise	8 weeks pre-treatment, then tumor inoculation	- Combined treatment: ↓ tumor volume, ↑ apoptosis, ↑ M1 macrophages, ↓ M2 macrophages - Suppressed JAK1/STAT6 *in vitro* - Prevented adipose tissue wasting via regulation of adipogenesis, lipolysis, and inflammation	(77)
BALB/C mice, 4T1 breast cancer	Daidzein (145 mg/kg) + exercise (15 m/min, 60 min/day)	42 days (20 days exercise pretreatment + 22 days treatment)	- Synergistic ↓ tumor growth - ↑ NK cell mobilization via epinephrine & IL-6 - Apoptosis via Fas/FasL mitochondrial pathway	(78)
Rats with DMH-induced colorectal cancer	Quercetin (50 mg/kg) + exercise training	12 weeks post-DMH	- ↓ tumor incidence - ↓ depressive-like behaviors - ↑ BDNF/TrKβ/β-catenin in prefrontal cortex - ↓ inflammation	(80)
Male Wistar rats with glioblastoma	Resistance-aerobic exercise + Nano-curcumin (80 mg/kg)	4 weeks; 3 days/week	- ↑ Wnt/β-catenin in tumor vs. healthy - Combined treatment ↓ Wnt/β-catenin most - Negative correlation of Wnt mRNA with musclin (r=-0.905)	(81)
Male Wistar rats with GBM	Aerobic exercise + nano-curcumin (80 mg/kg)	4 weeks; 3 days/week aerobic exercise, 5 days/week gavage	- Tumor ↑ TGF-β1, TRAF6, CTGF mRNA in myocardium - AE or AE + N-CUR ↓ TGF-β1, protective on heart	(82)
Male Wistar rats with GBM	Aerobic exercise (45 min/day, 5x/week) + green tea extract (1.34 mL, 3x/week)	8 weeks	- ↑ NF-kB in CCt - ↓ NF-kB with exercise ± extract - ↓ p53 in CTr, CEx, CTr+CEx - COX-2 unchanged	(79)

DEGs, Differentially Expressed Genes; HIIT, High-Intensity Interval Training; EC, Endurance exercise + Curcumin, E, Endurance exercise; CC, Curcumin alone; THQ, Tumor + HIIT + Quercetin, TH, Tumor + HIIT, T, Tumor control; AE, Aerobic Exercise, N-CUR, Nano-Curcumin, GEN, Genistein; DMH, 1,2-Dimethylhydrazine; CRC, Colorectal Cancer; GBM, Glioblastoma Multiforme; NK, Natural Killer cells; VEGF-A, Vascular Endothelial Growth Factor-A; TIE-2, Tyrosine Kinase with Immunoglobulin-like and EGF-like Domains 2; TNF-α, Tumor Necrosis Factor-alpha; NF-κB, Nuclear Factor kappa B; IL-4, Interleukin-4; STAT6, Signal Transducer and Activator of Transcription 6; ERK1/2, Extracellular Signal-Regulated Kinases 1/2; PI3K, Phosphoinositide 3-Kinase; IL-18, Interleukin-18; CTGF, Connective Tissue Growth Factor; TRAF6, Tumor Necrosis Factor Receptor-Associated Factor 6; TGF-β1, Transforming Growth Factor-beta 1; COX-2, Cyclooxygenase-2; p53, Tumor suppressor protein p53; BDNF, Brain-Derived Neurotrophic Factor; TrKβ, Tyrosine Kinase B receptor.

An experimental examined the interactive influences of aerobic exercise and curcumin supplementation on the progression of breast cancer through the TNF-α/NF-κB signaling pathway utilizing a 4T1 breast cancer murine model. Female BALB/c mice were allocated to groups for endurance training, curcumin treatment, a combined intervention, or a control condition over a duration of five weeks. Both aerobic exercise and curcumin administered independently markedly diminished tumor proliferation and intratumoral expression of TNF-α and NF-κB genes in comparison to the control group, whereas the combined intervention yielded a more pronounced inhibitory effect than exercise alone. These results exhibited that aerobic training synergistically amplifies the anti-inflammatory and anti-neoplastic properties of curcumin, resulting in the attenuation of TNF-α/NF-κB signaling and a decrease in breast cancer proliferation (75). Another work evaluated the synergistic effects of endurance training and curcumin on the advancement of breast cancer through the modulation of the IL-4/STAT-6 signaling pathway within a 4T1 breast cancer murine model. Female BALB/c mice were systematically assigned to exercise, curcumin, combined treatment, or control cohorts over a duration of five weeks. All experimental interventions resulted in a statistically significant reduction in tumor proliferation when compared to the control group. The combined regimen of endurance training and curcumin administration substantially diminished intratumoral IL-4 and STAT-6 gene expression, whereas endurance exercise alone led to reductions in both biomarkers, and curcumin administration singularly inhibited STAT-6 expression. Collectively, the results suggested that endurance training synergistically amplifies the anti-tumor properties of curcumin by attenuating IL-4/STAT-6 signaling, thereby facilitating a more pronounced suppression of breast cancer growth than either treatment modality in isolation (76). The study examined the oncological effects of the concurrent administration of genistein supplementation alongside regular moderate-intensity physical activity on breast cancer utilizing both *in vivo* and *in vitro* experimental models. Female BALB/c mice subjected to exercise either in isolation or in conjunction with genistein exhibited a delayed onset of tumors and a reduction in tumor proliferation when juxtaposed with the administration of genistein alone. The integrative intervention amplified apoptotic signaling pathways, fostered M1 macrophage polarization, and inhibited M2 macrophage polarization within breast tumor microenvironments. Correspondingly, *in vitro* exposure to genistein, along with myokines induced by exercise, resulted in the suppression of M2 macrophage markers and a downregulation of the JAK1/STAT6 signaling cascade. Furthermore, the combined therapeutic regimen alleviated adipose tissue depletion by modulating processes associated with adipogenesis, lipolysis, and systemic inflammatory responses. Collectively, these data demonstrated that the synergistic effects of genistein and moderate-intensity exercise effectively impede the progression of breast cancer through mechanisms involving apoptosis induction, immune system reprogramming, and metabolic regulation (77). Another study investigated the inhibition of daidzein or/and regular This investigation explored the synergistic influences of consistent physical activity and daidzein on the progression of breast cancer, as well as the underlying biological mechanisms in a 4T1 breast cancer murine model. BALB/c mice were subjected to a regimen of exercise training both prior to and subsequent to tumor implantation and were administered daidzein supplementation via gavage. Individually, both exercise and daidzein demonstrated inhibitory effects on tumor growth; however, their concomitant application resulted in a significantly enhanced suppressive outcome. From a mechanistic perspective, the combined intervention facilitated the mobilization and redistribution of natural killer cells through elevated levels of epinephrine and interleukin-6, as well as promoted the apoptosis of cancer cells through the activation of the Fas/FasL-mediated mitochondrial apoptotic pathway. These observations shown that the integration of regular exercise with daidzein produces synergistic anti-tumor effects and may constitute a promising non-pharmacological approach for the prevention and treatment of breast cancer (78).

### 6.2 Prostate cancer

While specific preclinical studies examining combined exercise and polyphenols in prostate cancer models are scarce, *in vitro* and animal evidence supports the independent benefits of each intervention. An experimental investigation assessed the impact of aerobic exercise training in conjunction with green tea extract supplementation on inflammatory and tumor-associated biomarkers in a chemically induced prostate cancer rat model. Adult male Wistar rats were subjected to an eight-week regimen of low-to-moderate intensity aerobic training, green tea extract administration, or a combination of both subsequent to the induction of prostate cancer. The presence of prostate cancer significantly elevated NF-κB levels in comparison to healthy control subjects, whereas aerobic training substantially decreased NF-κB expression in relation to cancer control and extract-only groups. Furthermore, exercise, green tea extract, and their synergistic application notably diminished p53 protein levels, whereas COX-2 expression exhibited no significant variation across the different experimental groups. Collectively, these findings indicated that sustained aerobic exercise, especially when integrated with green tea extract, may mitigate prostate cancer-associated inflammatory and tumor-related signaling pathways, thereby endorsing the viability of lifestyle-based interventions as adjunctive therapeutic strategies (79).

### 6.3 Gastrointestinal cancers

The body of work on polyphenol–exercise synergy in GI cancers is primarily conceptual or focused on single interventions. An investigation explored the synergistic effects of quercetin supplementation in conjunction with exercise training on depression associated with colorectal cancer in rats subjected to DMH-induced colorectal cancer. The administration of DMH precipitated tumor formation, the manifestation of depressive-like behaviors, neural impairment, and an elevation of inflammatory cytokines, concomitant with a decrease in the expression of BDNF, Trkβ, and β-catenin in the prefrontal cortex. The integrated application of quercetin and exercise markedly diminished tumor incidence, ameliorated depressive-like behaviors, attenuated inflammation, and enhanced the BDNF/Trkβ/β-catenin signaling pathway. These results elucidate that exercise collaborates with quercetin to manifest both anti-tumor and neuroprotective outcomes, underscoring a promising approach to mitigate cancer-related depression through central neurotrophic and signaling mechanisms (80).

### 6.4 Brain cancer

A study examined the impact of integrated resistance-aerobic exercise and nano-curcumin supplementation on the Wnt/β-catenin signaling pathway within a rat model of glioblastoma multiforme, alongside its correlation with the muscle-derived myokine musclin. Male Wistar rats, subjected to stereotactic induction of glioblastoma, were systematically allocated into exercise, nano-curcumin, combined intervention, or control cohorts for a duration of four weeks. The induction of tumors resulted in a significant elevation of Wnt/β-catenin expression in the brain, whereas both resistance-aerobic exercise and nano-curcumin, especially when administered in conjunction, substantially attenuated the activation of this signaling pathway in comparison to tumor controls. Importantly, a strong negative correlation was observed between Wnt mRNA expression and musclin mRNA levels in the tumor group receiving nano-curcumin, thereby suggesting the existence of crosstalk between muscle and brain. Collectively, these observations demonstrated that the synergistic application of exercise and nano-curcumin supplementation may inhibit glioblastoma-related Wnt/β-catenin signaling, potentially mediated through myokines induced by physical exercise (81). Another study evaluated the impact of aerobic exercise and nano-curcumin supplementation on cardiac TGF-β1/TRAF6 and CTGF signaling pathways in rats subjected to glioblastoma. Male Wistar rats were systematically allocated to distinct groups including healthy control, tumor control, aerobic exercise, nano-curcumin, or a combined intervention regimen for a duration of four weeks. The induction of brain tumors resulted in a statistically significant elevation in myocardial TGF-β1, TRAF6, and CTGF expression levels. The implementation of aerobic exercise, either in isolation or in conjunction with nano-curcumin, led to a marked decrease in TGF-β1 concentrations, thereby suggesting a protective role against cardiac remodeling induced by tumors. The results imply that aerobic exercise, especially when integrated with nano-curcumin, may alleviate cardiac fibrosis in rats harboring tumors through the modulation of TGF-β1/TRAF6 and CTGF signaling pathways, and further investigations are necessary to refine the optimal type and dosage of exercise (82).

## 7 Clinical evidence: Functional outcomes and physical performance

Emerging clinical evidence suggests that the integration of structured exercise regimens with polyphenol supplementation has the potential to improve functional outcomes and physical performance in individuals diagnosed with cancer and other chronic health conditions. Exercise interventions, encompassing aerobic, resistance, and hybrid modalities, have been consistently shown to enhance muscle strength, cardiorespiratory fitness, and overall physical functionality among cancer survivors, thereby alleviating cancer-related fatigue and preventing deconditioning (10, 83). Polyphenolic compounds, including curcumin, resveratrol, and catechins derived from green tea, have been explored as supplementary therapeutic agents owing to their anti-inflammatory, antioxidant, and metabolic regulatory effects. Despite the predominance of research concentrating on molecular and cellular outcomes, a number of clinical trials indicate possible enhancements in physical performance when polyphenols are administered in conjunction with exercise (84, 85). For example, supplementation with curcumin or resveratrol in combination with endurance or resistance training has been linked to improved muscle recovery, diminished exercise-induced inflammation, and enhancements in grip strength and walking endurance among older adults (86, 87). Moreover, the administration of polyphenols may enhance adaptations induced by exercise through the modulation of signaling pathways pertinent to muscle metabolism, mitochondrial functionality, and oxidative stress, such as the PI3K/Akt, AMPK, and NF-κB pathways (88). These molecular alterations result in significant functional enhancements, which encompass increased aerobic capacity, improved postural stability, and diminished impairments associated with sarcopenia. Notably, clinical investigations reveal that the combined application of exercise and polyphenol interventions is generally well-tolerated, exhibiting minimal adverse effects, thereby endorsing their viability as non-pharmacological approaches to maintain or augment physical function in susceptible populations, including individuals diagnosed with cancer, metabolic syndrome, and neurodegenerative conditions (83, 84). Nevertheless, the variability in study design, types of polyphenols utilized, dosages administered, and exercise protocols necessitates the execution of further large-scale, randomized clinical trials to ascertain optimal methodologies and clarify dose–response relationships for the attainment of maximal functional advantages.

## 8 Translational implications for rehabilitation

The temporal relationship between polyphenol consumption and physical exercise may significantly impact rehabilitation outcomes. Supplementation prior to exercise has the potential to bolster antioxidant defenses, mitigate oxidative stress resulting from exercise, and enhance endurance performance, whereas the administration of polyphenols subsequent to exercise may facilitate recovery through the modulation of inflammation and the promotion of muscle repair (85, 89). Customizing the timing of polyphenol intake in accordance with the nature and intensity of the exercise, the metabolic status of the patient, and the pharmacokinetic properties of polyphenols can optimize synergistic effects within clinical populations. The effective translation of clinical applications necessitates meticulous consideration of the dosage and bioavailability of polyphenols. Numerous polyphenolic compounds, such as curcumin and resveratrol, demonstrate limited oral bioavailability attributed to suboptimal absorption and rapid metabolic degradation (89, 90). Approaches such as nano-encapsulation, concurrent administration with bioenhancers, or the utilization of sustained-release formulations can enhance systemic bioavailability (91, 92). The determination of optimal dosing should strive for a balance between therapeutic efficacy and safety, while also being tailored to the patient’s age, body mass, and existing comorbidities.

Polyphenol supplementation is typically well-accepted; however, it is imperative to consider potential interactions with conventional cancer therapies. Certain polyphenols may modulate the activity of cytochrome P450 enzymes or impact oxidative stress pathways, thereby potentially influencing the efficacy or toxicity of chemotherapeutic agents (93–95). Existing evidence advocates for a cautious and monitored application of polyphenols in conjunction with cancer therapies, underscoring the necessity of consulting healthcare professionals and integrating supplementation within established therapeutic frameworks. The incorporation of polyphenol supplementation into personalized exercise rehabilitation programs necessitates a patient-centered methodology. The prescription of exercise should encompass considerations of frequency, intensity, type, and duration that are tailored to the individual patient’s functional capacity and disease progression (10, 96). The combination of polyphenols with structured aerobic, resistance, or mixed training modalities has the potential to enhance musculoskeletal recovery, mitigate inflammation, and elevate overall physical performance. Clinicians and rehabilitation professionals should diligently monitor functional outcomes, adjust exercise intensity based on individual tolerance levels, and synchronize supplementation strategies to optimize therapeutic synergy.

## 9 Limitations

Despite an increasing scholarly focus on polyphenol-based methodologies as supplementary interventions for musculoskeletal recovery and exercise rehabilitation in oncological patients, numerous limitations impede the robustness, consistency, and translational utility of the extant evidence. A fundamental obstacle is the variability inherent in study design. Clinical and preclinical investigations differ significantly in terms of polyphenol type, formulation, dosage, and methods aimed at enhancing bioavailability, in addition to the duration and timing of interventions in relation to exercise or therapeutic modalities. For example, research examining curcumin, resveratrol, or extracts abundant in flavonoids utilizes preparations that span from whole-food sources to highly standardized or nano-encapsulated supplements, thereby complicating inter-study comparisons. Another notable limitation is the prevalent dependence on surrogate molecular markers instead of clinically relevant functional outcomes. Numerous trials document reductions in inflammation, oxidative stress, or markers indicative of muscle damage; however, these alterations do not consistently result in quantifiable enhancements in physical performance, mobility, muscle strength, or recovery during cancer rehabilitation. This disjunction is especially pronounced in research endeavors that integrate polyphenols with structured physical activity, wherein beneficial biomarker modulation does not invariably correlate with augmented endurance, functional capacity, or quality-of-life indicators. The heterogeneity among participants further constrains the generalizability of findings. The majority of investigations concentrate on young or middle-aged populations, with scant representation of older cancer survivors, female patients, or individuals with comorbidities that are frequently associated with cancer treatment. Furthermore, the ecological validity of these studies is commonly low: exercise protocols are conducted under controlled conditions and supervision, polyphenol supplementation is meticulously timed, and interventions seldom align with real-world rehabilitation scenarios such as postoperative recovery, chemotherapy-induced fatigue, or variable functional status. Concerns regarding quality control and regulatory disparities remain paramount. Polyphenol supplements, particularly those derived from botanicals and algae, exhibit considerable variation in terms of purity, standardization, and bioactive constituents. Inconsistencies from batch to batch, insufficient labeling, and variations in extraction or encapsulation techniques may significantly impact both the efficacy and safety of these products. Patients undergoing oncological treatments may encounter increased risk owing to possible drug-nutrient interactions, thereby emphasizing the necessity for meticulous monitoring. Collectively, these constraints highlight the pressing need for methodologically rigorous, standardized, and clinically pertinent investigations to substantiate polyphenol-based interventions as viable adjuncts in the recovery of musculoskeletal function and exercise rehabilitation for individuals with cancer. Future inquiries should prioritize the integration of functional outcomes, the optimization of dosing and timing in relation to therapy and exercise, and the diversification of participant demographics to guarantee that translational recommendations are both safe and efficacious across varied patient populations.

## 10 Future directions and conclusion

Preclinical investigations using rodent cancer models consistently demonstrate that polyphenols such as curcumin, resveratrol, and genistein can enhance the beneficial effects of both aerobic and resistance exercise, contributing to tumor suppression, preservation of skeletal muscle mass, and modulation of key molecular pathways involved in inflammation, metabolism, and cancer progression. In contrast, the available clinical evidence remains limited, but emerging studies suggest that the combined use of polyphenolic compounds and structured exercise programs may improve muscular strength, endurance, and post-exercise recovery, particularly in individuals undergoing or recovering from oncological treatment. Although these findings are promising, further well-designed clinical trials are required to confirm their effectiveness and determine optimal supplementation and exercise strategies.

The studies reviewed in this manuscript collectively suggest that combining polyphenol supplementation with exercise may represent a promising adjunct strategy for improving cancer-related musculoskeletal outcomes, recovery capacity, and, in preclinical settings, tumor-related and muscle-preserving adaptations. Across the literature, the most consistent evidence comes from preclinical rodent models, in which polyphenols such as curcumin, resveratrol, and genistein enhanced the effects of exercise on tumor suppression, attenuation of muscle loss, and modulation of signaling pathways central to cancer progression and muscle remodeling, including Wnt/β-catenin, PI3K/Akt, TNF-α/NF-κB, IL-4/STAT6, and TGF-β1/TRAF6. These mechanistic findings are further supported by the broader biological framework discussed in the manuscript, which indicates that exercise-induced mechanotransduction and metabolic signaling may interact with polyphenol-mediated reductions in oxidative stress and inflammatory signaling, with possible convergence at intracellular nodes such as AMPK and PI3K/Akt.

In contrast, the clinical literature remains limited, heterogeneous, and more focused on exercise recovery than on cancer-specific endpoints. Nevertheless, the available human studies provide encouraging signals that some polyphenols may improve muscle soreness, aspects of functional recovery, strength preservation, and selected endurance-related outcomes when used alongside exercise. Importantly, however, these benefits are not uniform across all interventions or outcomes, and the inconsistency itself provides critical guidance for future study design. Among the polyphenols reviewed, curcumin is one of the best characterized in terms of formulation, dosing, and timing. The manuscript describes oral administration using bioavailability-enhanced preparations such as LipiSperse, Cureit™, and CurcuWIN^®^, as well as a turmeric drink. Reported human dosing strategies included 200 mg curcuminoids, 50 mg as a comparator dose, 180 mg/day, and 60 mL twice daily for the turmeric drink. Intervention duration varied substantially, ranging from acute peri-exercise administration, to 7 days before eccentric exercise, 4 days after exercise, 8 weeks, and repeated administration across 8 competitive matches in elite football players. This variability in design is important, because the reviewed findings suggest that curcumin’s effects may depend not only on dose, but also on timing relative to muscle-damaging exercise. Across these studies, curcumin most consistently reduced soreness and improved recovery-related measures, while also modulating IL-6, IL-10, TNF-α, hs-CRP, and in some studies creatine kinase. Some trials also reported preservation of isokinetic knee extension torque, flexion torque, and power at the 200 mg dose, and one study observed a mild improvement in VO_2_max. However, not all performance indices improved, suggesting that curcumin may exert stronger effects on post-exercise inflammation, muscle damage, and perceived recovery than on direct enhancement of performance capacity. Notably, one study indicated that post-exercise administration may be more effective than pre-exercise dosing, emphasizing the need for future trials to treat timing as a primary design variable rather than a secondary consideration.

The evidence for tart cherry is similarly promising but notably mixed. Tart cherry interventions were delivered orally in several forms, including powdered extract, spray-dried extract, concentrated tart cherry products, juice blends, tart cherry juice, and concentrate. Doses included 480 mg/day of Montmorency powdered extract, 1000 mg/day of concentrated tart cherry, 355 mL tart cherry juice twice daily, and 60 mL/day of tart cherry concentrate. Intervention durations ranged from 4 days, 8 days, and 10 days, to 7 days before competition plus race day, and up to 6 weeks. These studies were performed across markedly different exercise models, including eccentric elbow flexor protocols, high-volume back squats (10 × 10 at 70% 1-RM), concentric–eccentric leg extension, and long-distance relay running, as well as in a chronic knee pain setting. Such protocol diversity likely contributes to the inconsistency of the findings. Some studies reported meaningful benefits, including less soreness, reduced pain, and attenuation of strength loss, with one study showing 4% versus 22% strength loss compared with placebo after eccentric elbow flexion. Other studies observed favorable biochemical responses, including attenuated increases in creatinine, AST, ALT, and bilirubin, and more rapid normalization of lymphocyte counts. However, other trials found no benefit for strength loss, power, work, neuromuscular activation, soreness, pain, sleep, wellbeing, inflammatory markers, or biomechanics, especially in longer or different clinical contexts. The meta-analytic summary cited in the manuscript indicates an approximately 9% improvement in maximal isometric strength and reductions in IL-6 and IL-8, but no pooled effect on CK, CRP, TNF-α, or perceived soreness. Overall, tart cherry appears most likely to be beneficial when used around strenuous, muscle-damaging exercise, particularly when started several days before and continued after exercise, but the magnitude and reliability of benefit seem highly dependent on dose, formulation, exercise modality, training status, and target muscle group.

Taken together, the reviewed clinical studies indicate that the combination of exercise and polyphenols is biologically plausible and clinically promising, but not yet sufficiently standardized to support firm translational recommendations. A major issue across the literature is the wide variability in polyphenol type, formulation, dosage, route of administration, bioavailability enhancement, supplementation duration, and timing relative to both exercise and cancer therapy. This variability makes direct comparison across studies difficult and weakens the ability to define an optimal regimen. In addition, many studies continue to rely heavily on surrogate molecular or biochemical markers, even though improvements in these markers do not necessarily translate into better muscle strength, mobility, endurance, recovery, or quality of life. The literature is also limited by participant heterogeneity and underrepresentation, particularly of older cancer survivors, women, and patients with comorbidities, which reduces generalizability to real-world oncology rehabilitation settings. Furthermore, ecological validity remains a concern because many interventions are tested under highly supervised conditions with tightly controlled supplementation timing, which may not reflect routine clinical practice. Additional translational barriers include inconsistent supplement quality control, regulatory variation, and the possibility of drug–nutrient interactions during cancer treatment, all of which warrant careful consideration in future trials.

Future research should therefore move beyond simply asking whether polyphenols are beneficial and instead determine which polyphenol, at what dose, in which formulation, by which route, for how long, and at what time relative to exercise and anticancer therapy, provides clinically meaningful benefit. Based on the reviewed studies, this is especially important because current interventions range from short-term acute protocols to multi-week supplementation, and from capsules and enhanced oral formulations to juice-based preparations, with substantially different biological availability and outcome profile. Future randomized trials should prioritize standardized formulations, explicit reporting of dose and bioavailability characteristics, and predefined comparisons of pre-exercise versus post-exercise versus continuous supplementation strategies. They should also include clinically meaningful endpoints, such as muscle strength, physical function, fatigue, treatment tolerance, recovery time, and quality of life, rather than biomarker changes alone. In the oncology setting, future studies should also stratify participants by cancer type, treatment phase, age, sex, nutritional status, and baseline physical capacity, and should explicitly monitor safety, adherence, and interactions with concurrent therapies. Finally, greater integration of mechanistic and clinical designs will be essential to determine whether the signaling effects observed in preclinical models truly translate into improved rehabilitation outcomes in humans.

A key limitation of the current literature is the substantial heterogeneity across studies, which restricts the generalizability and clinical translation of findings. Existing investigations differ widely in polyphenol type, dosage, formulation, route of administration, supplementation duration, and timing relative to exercise, as well as in participant characteristics such as age, training status, health condition, and cancer-related variables. This variability complicates direct comparisons between studies and makes it difficult to identify optimal supplementation strategies. Furthermore, much of the mechanistic evidence supporting the synergistic effects of polyphenols and exercise originates from preclinical models, where consistent improvements in tumor-related signaling pathways and muscle preservation have been observed. In contrast, human clinical trials remain relatively limited and often report inconsistent outcomes, with many studies focusing primarily on short-term recovery markers or biochemical indicators rather than clinically meaningful functional endpoints. Together, these limitations highlight the need for well-designed, adequately powered clinical trials using standardized dosing protocols, clearly defined formulations, and clinically relevant outcome measures to better determine the efficacy and translational potential of combined polyphenol supplementation and exercise interventions.

In conclusion, the evidence reviewed here supports the view that polyphenols may enhance selected exercise-related benefits, with the strongest support currently seen in preclinical models and more limited but encouraging evidence in human studies. Curcumin appears to show the most consistent effects on soreness reduction, inflammatory modulation, and preservation of some aspects of muscle function, especially when bioavailable oral formulations are used and timing is optimized. Tart cherry also shows potential, particularly for reducing soreness and limiting strength loss after damaging exercise, although findings are more variable and appear highly sensitive to formulation and protocol differences. More broadly, the reviewed preclinical data indicate that polyphenol–exercise combinations may influence both muscle preservation and tumor-related biology through pathways such as Wnt/β-catenin, PI3K/Akt, TNF-α/NF-κB, IL-4/STAT6, and TGF-β1/TRAF6 (*PCan.docx*, p. 3). However, the field is not yet mature enough to define universal clinical recommendations. At present, the literature is best interpreted as demonstrating a promising but still developing adjunctive strategy for cancer rehabilitation. To fulfill this promise, future work must adopt more rigorous, standardized, and clinically relevant approaches that define precise regimens for dose, route of administration, duration, and timing, while also evaluating real-world feasibility, safety, and meaningful patient outcomes.

Future research should prioritize the development of standardized polyphenol preparations with clearly defined composition, dosage, and bioavailability to improve comparability across studies and facilitate clinical translation. In parallel, there is a need to refine exercise protocols specifically tailored to cancer rehabilitation, taking into account factors such as treatment stage, functional capacity, and individual patient needs. Upcoming studies should also place greater emphasis on clinically meaningful outcomes, including muscle strength, endurance, functional performance, fatigue, and quality of life, rather than relying primarily on biochemical or molecular markers. Ultimately, well-designed and adequately powered clinical trials will be necessary to generate the evidence required to establish clear, evidence-based guidelines for integrating polyphenol supplementation into cancer rehabilitation programs, ensuring both safety and efficacy in clinical practice.
